# Correlation between frailty and reduction in cortical thickness in patients with chronic obstructive pulmonary disease

**DOI:** 10.1038/s41598-024-53933-0

**Published:** 2024-03-13

**Authors:** Ayumi Fukatsu-Chikumoto, Tsunahiko Hirano, Shun Takahashi, Takuya Ishida, Kasumi Yasuda, Tomohiro Donishi, Kazuyoshi Suga, Keiko Doi, Keiji Oishi, Shuichiro Ohata, Yoriyuki Murata, Yoshikazu Yamaji, Maki Asami-Noyama, Nobutaka Edakuni, Tomoyuki Kakugawa, Kazuto Matsunaga

**Affiliations:** 1 Department of Respiratory Medicine and Infectious Disease, Graduate School of Medicine, Yamaguchi University, 1-1-1 Minami-Kogushi, Ube, 755-8505 Japan; 2 Department of Psychiatry, Osaka University Graduate School of Medicine, Suita, 565-0871 Japan; 3 Department of Neuropsychiatry, Wakayama Medical University, Wakayama, 641-0012 Japan; 4 Graduate School of Rehabilitation Science, Osaka Metropolitan University, Habikino, 583-8555 Japan; 5 Clinical Research and Education Center, Asakayama General Hospital, Sakai, 590-0018 Japan; 6 Department of System Neurophysiology, Wakayama Medical University, Wakayama, 641-0012 Japan; 7 Department of Radiology, St. Hill Hospital, Ube, 755-0155 Japan; 8 Department of Pulmonology and Gerontology, Graduate School of Medicine, Yamaguchi University, Ube, 755-8505 Japan

**Keywords:** Health care, Neurology

## Abstract

Physical inactivity and cognitive impairment in patients with chronic obstructive pulmonary disease (COPD) can lead to frailty and poor prognoses. However, little is known regarding the association between frailty and the human brain. We hypothesized that the brain structure could change according to frailty in patients with COPD and focused on cortical thickness. Cortical thickness measured by magnetic resonance imaging and frailty scores using the Kihon Checklist (KCL) were assessed in 40 patients with stable COPD and 20 healthy controls. Among the 34 regions assessed, multiple regions were thinner in patients with COPD than in healthy individuals (*p* < 0.05). We found significant negative correlations between the eight regions and the KCL scores only in patients with COPD. After adjusting for age and cognitive impairment, the association between the left and six right regions remained statistically significant. The correlation coefficient was the strongest in the bilateral superior frontal gyrus (left: ρ = − 0.5319, *p* = 0.0006) (right: ρ = − 0.5361, *p* = 0.0005). Interestingly, among the KCL scores, the daily activity domain showed the strongest correlation (sensitivity, 90%; specificity, 73%) with the bottom quartile of the reduction in the superior frontal gyrus. Frailty in patients with COPD is associated with a thickness reduction in the cortical regions, reflecting social vulnerability.

## Introduction

Physical inactivity and cognitive impairment in patients with chronic obstructive pulmonary disease (COPD) can lead to frailty, which is associated with poor prognosis^1,2^. The poor prognosis is due to the correlation between frailty and an increased risk of adverse outcomes, including falls, hospitalization, and mortality^3^. Physical inactivity has also been reported to increase inflammatory cytokines, causing chronic inflammatory responses that lead to systemic-dependent diseases such as diabetes, atherosclerosis, dementia, and cancer, which are associated with poor prognosis^4–7^.

The prevalence of frailty in patients with COPD is reported to be at 20–57%^8^. Our previous study showed that patients with COPD had a higher proportion of individuals with overlapping sedentary behaviors and cognitive impairment (motoric cognitive risk [MCR]) than those with asthma or healthy individuals^9^. This suggests that the poor prognosis of COPD may be associated with a link between frailty and the brain.

COPD also induces a frailty cycle accompanied by MCR^8^. This vicious cycle of physical inactivity might lead to sarcopenia, which in turn leads to sedentary behaviors that avoid exercise due to dyspnea, resulting in decreased quality of life (QOL)^8^. However, little is known about the association between this pathophysiology on the brains of patients. Previous attempts have been made to use brain imaging techniques to reveal the brain structures of patients with COPD. To date, stable, non-hypoxemic COPD has been reported to have decreased white matter integrity throughout the brain and widespread impairment of gray matter function activation^10^. We investigated the association between the COPD frailty pathology and brain structure in detail. We previously found that frailty was associated with reduced hippocampal volume as measured using magnetic resonance imaging (MRI) in patients with COPD^11^.

Therefore, we hypothesized that frail patients with COPD may exhibit structural changes in other areas of the brain in addition to hippocampal atrophy. We aimed to evaluate the relationship between frailty and cortical thickness in patients with COPD. Moreover, we aimed to determine the daily living conditions associated with the reduction in cortical thickness.

## Results

### Characteristics of the participants

As shown in Table 1, 40 patients with COPD and 20 healthy controls were recruited from the Yamaguchi Medical University Hospital. The COPD group included more male patients and older patients than that of the healthy group. In our study, although 90% of the patients with COPD were classified as having Global Initiative for Chronic Obstructive Lung Disease (GOLD) stage I/II, approximately 80% had frailty as assessed using the Kihon Checklist (KCL)^12,13^ or mild cognitive impairment (MCI) status^14,15^. In addition, patients with COPD had reduced physical activity (exercise [EX]) compared with healthy participants.

**Table 1:** Characteristics of the participants.

Measure	Healthy (n = 20)	COPD (n = 40)	*p*
Sex (male/female)	7/13	39/1	< 0.0001
Age (years) (Mean ± S.D.)	61.3 ± 9.58	70.6 ± 8.21	0.0013
BMI (kg/m^2^) (Mean ± S.D.)	22.60 ± 3.35	23.26 ± 3.29	0.4902
Smoking status (Cu/Ex/Non)	1/5/14	12/28/0	< 0.0001
Pack years (Mean ± S.D.)	9.75 ± 16.11	46.60 ± 25.49	< 0.0001
%VC (%) (Mean ± S.D.)	105.67 ± 12.10	97.00 ± 18.67	0.0790
%FVC (%) (Mean ± S.D.)	106.62 ± 16.37	98.70 ± 19.26	0.1263
%FEV1.0 (Mean ± S.D.)	104.30 ± 11.65	73.96 ± 18.07	< 0.0001
%DLco/VA (%) (Healty = 6) (Mean ± S.D.)	94.53 ± 9.15	77.84 ± 24.09	0.0728
GOLD (1/2/3/4)	–	15/21/4/0	
Kihon Checklist (KCL) (Mean ± S.D.)	3.4 ± 2.23	8.3 ± 5.08	0.0001
KCL (Non-frail/Pre-frail/Frail)	9/10/1	9/11/20	0.0008
MoCA-J score	26.55 ± 2.52	23.10 ± 3.22	< 0.0001
MoCA-J score ≦ 25	2	31	< 0.0001
EX (Mean ± S.D.)	5.83 ± 2.80	2.60 ± 2.11	< 0.0001

*COPD:* chronic obstructive pulmonary disease, *GOLD:* Global Initiative for Chronic Obstructive Lung Disease, *S.D.:* standard deviation, *MoCA-J:* Japanese version of the Montreal Cognitive Assessment, *BMI*: body mass index, *Cu:* current smoker, *Ex:* ex-smoker, *Non:* non-smoker, *VC:* vital capacity, *FVC:* forced vital capacity, *FEV1:* forced expiratory volume in 1 s, *DLco:* diffusing capacity of lung carbon monoxide, *VA:* alveolar volume, *KCL:* Kihon Checklist, *EX:* exercise.

### Comparison of cortical thickness between Patients with COPD and healthy participants

**Figure 1:**
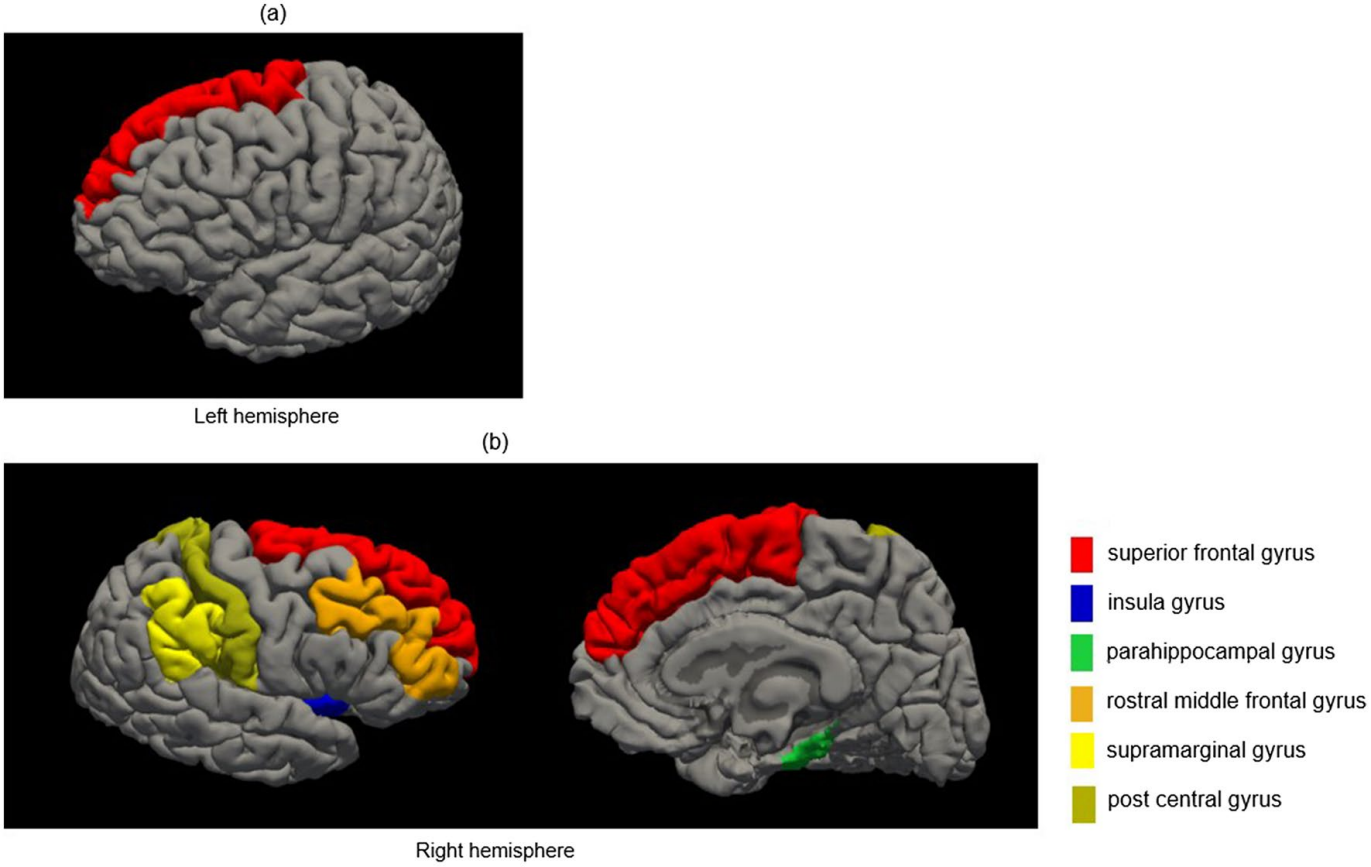
Frailty specific cortical thickness reduction area in patients with COPD. In the partial correlation between frailty and cortical thickness with age as a covariate, areas specific to patients with COPD are shown in this figure. Each cortical area is highlighted in a different color. (**a**) left and (**b**) right hemispheres.

**Figure 2:**
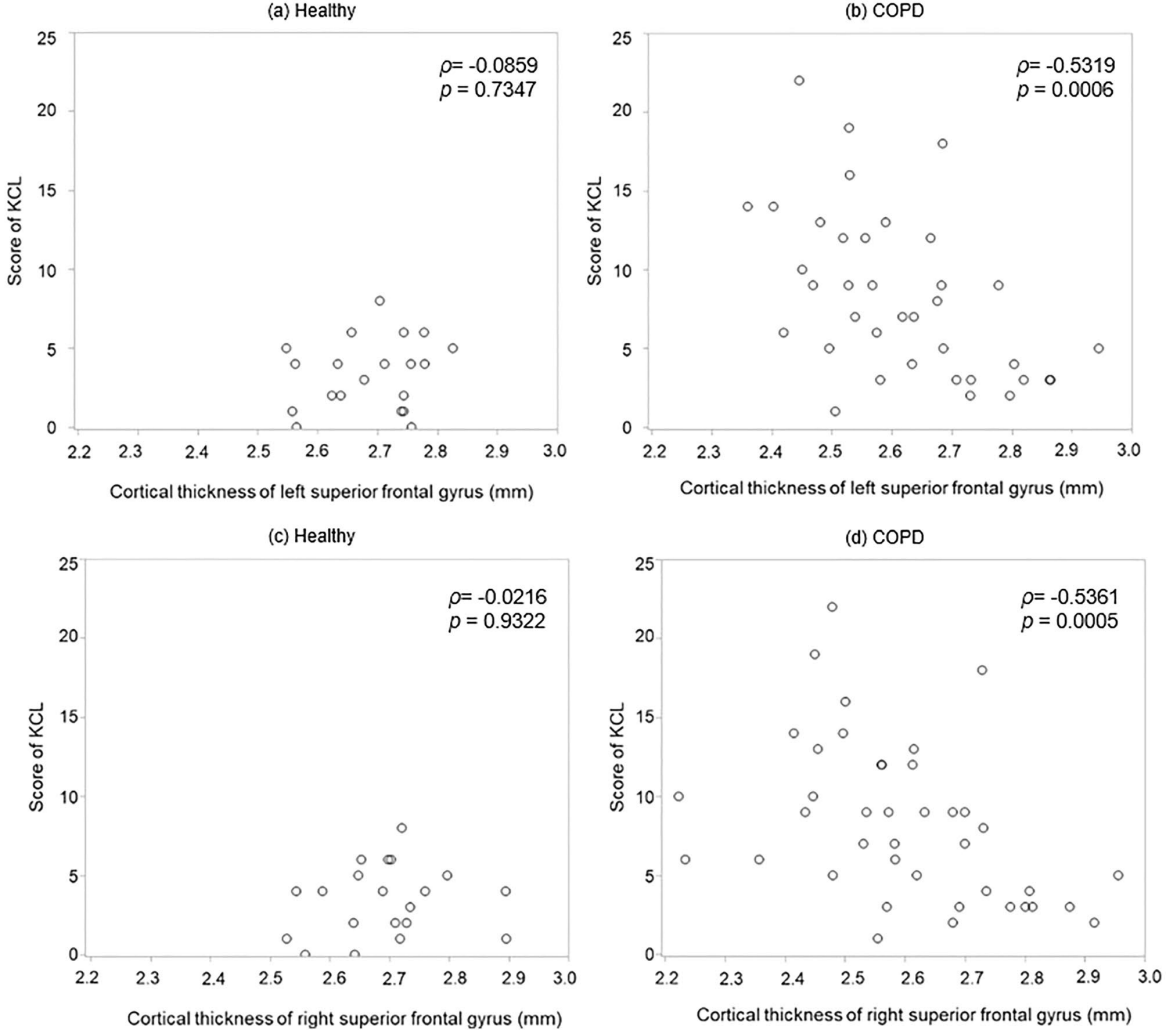
Frailty and cortical thickness reduction in the superior frontal gyrus. A scattergram of the association between the KCL score and cortical thickness of the superior frontal gyrus. (**a**) left side in the healthy, (**b**) left side in COPD, (**c**) right side in the healthy, (**d**) right side in COPD.

Of the 34 cortical regions on each side, 14 on the right side and 12 on the left side were significantly thinner in the COPD group than in the healthy group (*p* < 0.05) (Table 2). We evaluated the association between these regions and KCL scores. We found eight regions with significant negative correlations in patients with COPD, but not in healthy individuals. The eight regions were the left superior frontal gyrus, left rostral middle frontal gyrus, right parahippocampal gyrus, right rostral middle frontal gyrus, right superior frontal gyrus, right supramarginal gyrus, right postcentral gyrus, and right insular gyrus (*p* < 0.05) (data not shown). Even when adjusted for age as a covariate, or for age and cognitive impairment, one region on the left and six regions on the right showed significant correlations (Supplementary Table S1a online, Table 3). The regions included the left superior frontal, right parahippocampal, right rostral middle frontal, right superior frontal, right supramarginal, right postcentral, and right insular gyri (*p* < 0.05). Each cortical region is colored as shown in Fig. 1. The correlation coefficient between the bilateral superior frontal gyrus and frailty score was the highest (left: ρ = − 0.5319, *p* = 0.0006) (right: ρ = − 0.5361, *p* = 0.0005). This correlation remained significant even after adjusting for BMI (Supplementary Table S1b online). Scatter plots of the relationship between the cortical thickness of the left and right superior frontal gyri and the KCL scores in each group are shown in Fig. 2.

**Table 2:** Comparison of cortical thickness between patients with COPD and healthy controls. The cortical thickness is 34 areas on each side, but COPD-specific thin cortical areas are extracted and noted.

Cortical thickness (mm)	Healthy (n = 20)		COPD (n = 40)		*p*
	Mean	S.D	Mean	S.D	
Caudal anterior cingulate					
R	2.655	0.19	2.470	0.25	0.0042
L	2.612	0.23	2.613	0.26	0.7958
Caudal middle frontal					
R	2.480	0.10	2.394	0.16	0.0220
L	2.493	0.12	2.407	0.16	0.0528
Inferior parietal					
R	2.419	0.09	2.311	0.15	0.0035
L	2.376	0.09	2.312	0.13	0.0423
Lateral occipital					
R	2.148	0.08	2.100	0.12	0.0305
L	2.061	0.09	2.051	0.15	0.8940
Medial orbitofrontal					
R	2.600	0.09	2.487	0.17	0.0101
L	2.501	0.12	2.402	0.16	0.0135
Para hippocampal					
R	2.698	0.22	2.516	0.30	0.0299
L	2.790	0.24	2.627	0.39	0.0620
Pars opercularis					
R	2.514	0.11	2.462	0.17	0.1363
L	2.533	0.08	2.448	0.14	0.0092
Pars orbitalis					
R	2.682	0.18	2.585	0.22	0.0933
L	2.729	0.15	2.570	0.22	0.0034
Pars triangularis					
R	2.403	0.10	2.348	0.13	0.0588
L	2.463	0.10	2.356	0.15	0.0046
Postcentral					
R	1.991	0.09	1.915	0.12	0.0447
L	1.981	0.09	1.925	0.11	0.0509
Precentral					
R	2.448	0.16	2.371	0.18	0.0385
L	2.480	0.10	2.359	0.19	0.0043
Rostral middle frontal					
R	2.346	0.09	2.263	0.12	0.0077
L	2.350	0.06	2.27	0.14	0.0132
Superior frontal					
R	2.693	0.10	2.603	0.17	0.0254
L	2.688	0.08	2.591	0.18	0.0132
Superior temporal					
R	2.726	0.10	2.626	0.16	0.0127
L	2.704	0.12	2.562	0.17	0.0008
Spramarginal					
R	2.454	0.09	2.351	0.14	0.0028
L	2.456	0.08	2.372	0.15	0.0103
Frontal pole					
R	2.687	0.20	2.625	0.28	0.1491
L	2.799	0.17	2.632	0.25	0.0052
Temporal pole					
R	3.737	0.20	3.53	0.35	0.0161
L	3.658	0.27	3.538	0.37	0.0918
Insula					
R	2.934	0.09	2.804	0.20	0.0015
L	2.910	0.12	2.817	0.20	0.0407

*COPD:* chronic obstructive pulmonary disease, *SD:* standard deviation, *R:* right, *L:* left.

**Table 3:** Partial correlation between frailty and cortical thickness with age and the MoCA-J score as covariates.

	Healthy (n = 20)		COPD (n = 40)		
	KCL				
	*ρ*	*p*	*ρ*		*p*
**Left**					
Superior frontal	− 0.0859	0.7347	− 0.5319		0.0006
**Right**					
Superior frontal	− 0.0216	0.9322	− 0.5361		0.0005
Insula	0.2861	0.2497	− 0.4933		0.0017
Para hippocampal	0.1360	0.5905	− 0.4474		0.0049
Post central	− 0.2758	0.2679	− 0.3358		0.0393
Rostral middle frontal	0.0826	0.7447	− 0.3475		0.0326
Supra marginal	0.4632	0.0529	− 0.3438		0.0346

*COPD:* chronic obstructive pulmonary disease, *KCL:* Kihon Checklist, *MoCA-J:* Japanese version of the Montreal Cognitive Assessment, *ρ:* partial correlation coefficient.

### Examination of cortical thickness and physical activity

We examined the relationships between the seven cortical regions identified in the previous paragraph and physical activity. As shown in Supplementary Table S2a, there was a significant correlation between the EX and cortical thickness in four of the seven cortical regions in the COPD group. When the correlation between cortical thickness and KCL was corrected by adding EX to age, MoCA-J score, and BMI, a significant correlation was found in four of the seven regions (Supplementary Table S2b).

### KCL sub-domain score associated with cortical thinning

We evaluated the relationship between the life conditions and reduction in cortical thickness in the COPD group. We focused on the superior frontal gyrus, which had the highest correlation coefficient. For the purposes of this study, we defined the group of patients that fall into the lowest 25% in cortical thickness as the "cortical thinning.”

**Figure 3:**
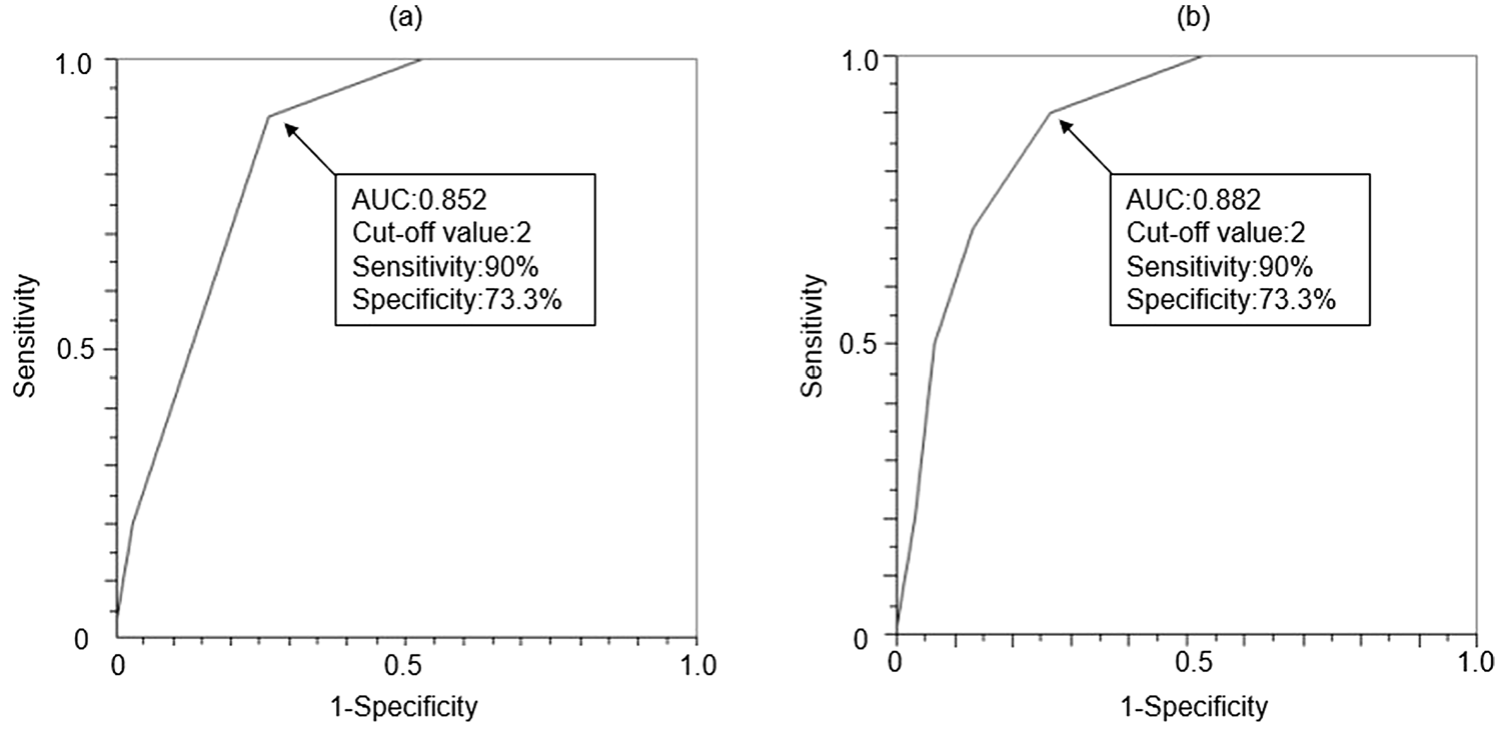
Utility of daily activity domain to capture cortical thickness reduction. Receiver operating characteristic (ROC) curves were plotted to evaluate the usefulness of KCL1-5 scores in detecting cortical thickness reduction in the superior frontal gyrus. (**a**) left and (**b**) right.

First, when the KCL domain was divided into 1–20 (unrelated to depression) and 21–25 (related to depression) questions, thickness reduction in the superior frontal gyrus was significantly associated with the 1–20 question domains (Supplementary Fig. S1). Notably, the question domain, including daily activities (KCL1-5), showed the strongest correlation with thickness reduction (Supplementary Fig. S2), with a sensitivity of 90% and specificity of 73%, capturing the bottom quartile in the superior frontal gyrus if two of the five questions were applicable (Fig. 3, left: area under the curve (AUC) = 0.85, right: AUC = 0.88). A comparison of the sensitivity and specificity between different KCL 1–5 score points also showed that two points comprised the cut-off value that was the best for screening (Table 4, Supplementary Fig. S3).

**Table 4:** Prediction of cortical thickness reduction in (a) the left superior frontal gyrus and (b) the right using KCL (1–5) score points.

Cut-off point	Sensitivity (%)	Specificity (%)	PPV (%)	NPV (%)
(a)				
1	100.0	46.7	38.5	100.0
2	90.0	73.3	52.9	95.7
3	60.0	83.3	54.5	86.2
4	40.0	90.0	57.1	81.8
5	20.0	96.7	66.7	78.4
(b)				
1	100.0	46.7	38.5	100.0
2	90.0	73.3	52.9	95.7
3	70.0	86.7	63.6	89.7
4	50.0	93.3	71.4	84.8
5	20.0	96.7	66.7	78.4

*KCL:* Kihon Checklist, *PPV:* positive predictive value, *NPV:* negative predictive value.

## Discussion

This study showed that frailty in patients with COPD is associated with a reduction in cortical thickness in specific brain regions. This correlation was the most pronounced in the superior frontal group. Moreover, domains reflecting disability in basic daily living activities in the KCL could be useful tools for capturing the structural brain signatures of frailty (brain frailty).

Structural abnormalities of the cortex are associated with several diseases and conditions^16–19^. Neuropsychiatric diseases cause structural changes in the brain; schizophrenia and Parkinson’s disease are associated with frailty^20,21^. There have been scattered reports that exercise can improve cortical thinning in patients with schizophrenia^22^ and that cortical thickness can predict motor responsiveness in patients with Parkinson's^23^. Based on these findings, we aimed to evaluate the relationship between frailty and cortical thickness in patients with COPD. Studies have demonstrated a relationship between structural changes in the brain and frailty. Most studies have reported that an increase in white matter lesions is associated with the prevalence of frailty^24^. The mechanism in these reports was based on the damage to blood vessels as a result of atherosclerosis. Moreover, it has been argued that exposure to risk factors such as smoking, hypertension, diabetes, and middle age accelerate atherosclerosis^25^. Similarly, in patients with COPD, there have been reports of deterioration in the cerebral structure, consistent with ischemic pathology^26–30^, although its relationship with frailty is unclear. It has been suggested that COPD-related deterioration in brain structure would occur secondary to small-vessel cerebrovascular disease^31^ due to many vascular risk factors associated with aging or COPD itself, as it is also a risk factor^30^. However, there are few reports on the association between cortical thickness reduction and frailty. Lu et al. clarified that 484 older adults aged 70 years and above with greater global cortical thickness were less likely to be pre-frail and frail at baseline^32^. Iritani et al. reported that frailty assessment using the KCL is useful in assessing frailty-related cerebral atrophy areas^33^. To the best of our knowledge, only one study has reported cortical thickness in patients with frail COPD^29^. The study compared cortical thickness with physical activity in 10 patients with COPD who underwent MRI and found that reduced cortical thickness had a significantly higher modified Medical Research Council dyspnea (mMRC) scale and fewer steps per day counted by the pedometer^29^. Patients with thinner cortices exhibited more dyspnea and less activity. In our study, the majority of the cortex that was thinned in COPD and was significantly correlated with frailty also showed a significant correlation with physical activity (EX). This is consistent with the results of previous reports^34,35^. Furthermore, even after adjusting for physical activity, a significant correlation was observed between frailty and cortical thickness. This suggests that frailty is related not only to physical activity, but also to factors other than activity (social, psychological, etc.), which will be discussed later.

We have previously revealed that the frailer the patients with COPD, the greater the reductions in the left and right hippocampal volumes^11^. In addition, the volume of the left hippocampal CA1 region was significantly correlated with QOL, indicating that hippocampal atrophy and decreased QOL may be associated with frailty in patients with COPD^11^. The KCL assesses the activities of daily living, which also reflect QOL. The present study showed that not only a decrease in hippocampal volume, but also a decrease in cortical thickness, a brain structural change, occurred between frailty and reduced QOL in patients with COPD. These findings suggest that the pathology of frailty in patients with COPD is related to structural changes in a wide range of brain regions.

We showed that a specific reduction in cortical thickness in the frailty phenotype among patients with COPD could occur independently of aging and cognitive function. The cortical thickness decreases with age. A study of 207 healthy adults reported a − 4.0 ± 0.76% change over time in the superior frontal gyrus^36^. Cortical thickness has also been associated with cognitive impairment. Blumen et al. showed that cortical thickness was lower in individuals with motor cognitive risk syndrome^37^. Moreover, there is a bidirectional relationship between physical frailty and cognitive impairment^38^. However, even after adjusting for age and the MoCA-J score, a significant correlation was found between cortical thinning and frailty in patients with COPD. This suggests that thinning of the brain cortex in patients with frail COPD is associated with various factors, such as lifestyle and environment, beyond the typical aging process.

However, the mechanisms underlying the reduction in cortical thickness in patients with frail COPD remain unknown. In addition to age and cognitive function, a correlation between smoking and reduction in cortical thickness has been reported^39,40^. All the patients with COPD in this study had a history of smoking. However, even when adjusted for pack-years, the bilateral superior frontal gyrus, which had the strongest correlation with frailty score, remained significant (data not shown). Although not examined in this study, tobacco smoke-induced oxidative stress may have partially affected the cortical thickness reduction. Respiratory function differed between COPD and healthy groups. In this study, five of the brain cortices, including the bilateral superior frontal gyrus, had a significant correlation with KCL, even after adjusting for % forced expiratory volume in 1 s (% FEV1) and age (data not shown). However, a previous study evaluated the relationship between cortical thickness reduction and respiratory function, and no association between forced vital capacity (FVC) and FEV1 was observed^41^. Therefore, further studies are warranted. The mechanism of the reduction in cortical thickness has been suggested to involve a combination of various factors, such as hypoxia, chronic inflammation, and oxidative stress.

The strength of this study is that it clarified the specific question domains in the KCL that are associated with brain frailty in patients with COPD. Although a previous study showed a relationship between physical inactivity and brain structure, the relationship between the level of daily living activities and brain structure has not been revealed^32,37,38^. In the present study, the thickness reduction in the bilateral superior frontal gyrus was most strongly correlated with the question domain, including daily activities (KCL1-5). These results were found regardless of age or cognitive impairment, indicating that difficulties in activities related to daily living are not simply due to the effects of aging or cognitive impairment, but also reflect social vulnerability. The superior frontal gyrus, which is associated with social vulnerability, is a brain region associated with various functions including cognition, attention, and depression^42^. As there is a cortical-subcortical linkage^43,44^, thinning of certain brain cortices in COPD may also affect the subcortex and consequently affect daily living. Interestingly, two of the five questions were applicable to the question domain, including daily activities, with a sensitivity of 90% and specificity of 73%, to assess cortical thickness reduction in the superior frontal gyrus. In summary, this subdomain is considered useful for screening for brain frailty. Although the direct relationship with reduced daily living activities is not clear, if patients with brain frailty are identified by earlier screening tests, supporting them in enabling the KCL1-5 items may easily improve their brain frailty. This can lead to early detection of frailty and early intervention in patients who were not previously eligible for treatment. This is consistent with the fact that most patients with COPD in the GOLD I/II phase were included in this study. Various preventive care programs have been reported to reduce frailty and improve the QOL in older adults^45^. The usefulness of exercise programs for local older adults in reflecting on their life activities and setting personalized life goals has also been reported^46^. To improve brain frailty in patients with COPD, in addition to these interventions, it may be necessary to provide social support, as KCL1-5 includes social behaviors such as visiting friends’ homes and counseling family and friends.

Our study had several limitations. The study was conducted at a university hospital, and it was difficult to recruit healthy elderly individuals as controls. Therefore, the number of participants in the control group was lower than that in the COPD group. There were also significant differences in age and sex between the groups. Similar to age, sex differences have been reported to be associated with cortical thickness^47–49^. Although we consider that there is an effect of sex differences in this study in correlation between cortical thickness and KCL, when adjusted for sex and in addition to the aforementioned age, MOCA-J scores, and BMI, significant partial correlations were found in four out of seven regions in the COPD group (Supplementary Table S3). Although some reports emphasize the relationship between sex differences and BMI^50^, regarding structural changes in brain cortical thickness in the COPD group, the correlation between cortical thickness and KCL was significant even when adjusted for BMI (Supplementary Table S1b). Strictly speaking, this study did not determine whether the relationship between frailty and cortical thickness is specific to COPD. This is because the COPD group was significantly more frail than the healthy participants. The control group should have included frail patients without COPD. However, this was difficult to achieve in this study. Another limitation of this study is that comorbidities in patients with COPD may affect frailty and cortical thickness. However, this aspect has not yet been evaluated. This study included a small number of patients with severe COPD. Moreover, this was a cross-sectional study. Since the causal relationship between cortical thinning and decreased physical activity is unclear, a longitudinal study with a larger sample size is necessary in the future.

## Conclusion

Frailty in patients with COPD is associated with a reduced thickness of specific cortical regions. It also reflects social vulnerability. Using KCL may lead to the early detection and intervention of brain frailty.

## Methods

### Participants

Forty patients with COPD and 20 healthy controls from the Yamaguchi Medical University Hospital were included in this study (Table 1). All patients were aged > 40 years and were diagnosed by a pulmonologist. All patients were in a stable condition with treatment based on the GOLD guidelines, without exacerbations, for at least 3 months prior to the study. Healthy controls were also over 40 years of age, defined as those who had no apparent respiratory disease despite a visit to a respiratory and infectious disease physician or who underwent positron emission tomography-computed tomography (PET-CT) screening and had no apparent respiratory disease.

This study was conducted in accordance with the ethical standards of the Declaration of Helsinki. This study was approved by the Ethics Committee of Yamaguchi University (June 6, 2016; Institutional Review Board No. H28-031) and Wakayama Medical University (August 19, 2016) and registered at the UMIN Clinical Trials Registry: UMIN000024645 (October 13, 2016). Informed consent was obtained from all the patients.

### Clinical assessments

Frailty was defined according to KCL scores^12,13^. There are various methods of assessing frailty. KCL is a self-reported checklist being one of them and was designed by a study group from the Japanese Ministry of Health, Labor and Welfare^12,13^. As shown in Table 5, 25 checklists assessed multiple aspects of frailty. It is divided into the following seven domains, each based on the intent of the question: daily living (1–5), motor functions (6–10), undernutrition (11, 12), oral functions (13–15), shut-in (16, 17), dementia (18–20), and depression (21–25)^51^. Difficulty in answering any question was counted as a score, and frailty was defined as a total score of eight or more, pre-frailty as four or more, and health as three or less. MCI was assessed using the Japanese version of the Montreal Cognitive Assessment (MoCA-J). Those with scores ≤ 25 on the MoCA-J were defined as having MCI. The MoCA-J generates trained rater scores (0–30) and assessed nine domains of cognition: attention, concentration, executive functions, memory, language, visuoconstructional skills, conceptual thinking, calculations, and orientation. It is considered a useful screening tool for assessing MCI^14,15^.

**Table 5:** Kihon Checklist (KCL).

No	Questions	Answer	
1	Do you go out by bus or train by yourself?	0.YES	1.NO
2	Do you go shopping to buy daily necessities by yourself?	0.YES	1.NO
3	Do you manage your own deposits and savings at the bank?	0.YES	1.NO
4	Do you sometimes visit your friends?	0.YES	1.NO
5	Do your family or friends turn to you for advice?	0.YES	1.NO
6	Do you normally climb stairs without using handrail or wall for support?	0.YES	1.NO
7	Do you normally stand up from a chair without any aids?	0.YES	1.NO
8	Do you normally walk continuously for 15 min?	0.YES	1.NO
9	Have you experienced a fall in the past 6 months?	1.YES	0.NO
10	Do you have a fear of falling while walking?	1.YES	0.NO
11	Have you lost 2 kg or more in the past 6 months?	1.YES	0.NO
12	Height: cm, BMI: kg/m^2^ If BMI is less than 18.5, this item is scored	1.YES	0.NO
13	Do you have any difficulties eating tough foods compared to 6 months ago?	1.YES	0.NO
14	Have you choked on your tea or soup recently?	1.YES	0.NO
15	Do you often experience having a dry mouth?	1.YES	0.NO
16	Do you go out at least once a week?	0.YES	1.NO
17	Do you go out less frequently compared to last year?	1.YES	0.NO
18	Do your family or your friends point out your memory loss? e.g. “You ask the same question over and over again.”	1.YES	0.NO
19	Do you make a call by looking up phone numbers?	0.YES	1.NO
20	Do you find yourself not knowing today’s date?	1.YES	0.NO
21	In the last 2 weeks have you felt a lack of fulfilment in your daily life?	1.YES	0.NO
22	In the last 2 weeks have you felt a lack of joy when doing the things you used to enjoy?	1.YES	0.NO
23	In the last 2 weeks have you felt difficulty in doing what you could do easily before?	1.YES	0.NO
24	In the last 2 weeks have you felt helpless?	1.YES	0.NO
25	In the last 2 weeks have you felt tired without a reason?	1.YES	0.NO

*BMI* body mass index.

Adapted with permission from John Wiley and Sons.

### MRI measures and neuroimaging analysis

Both the COPD and healthy groups underwent head MRI within 28 days of enrolment. Data were acquired from a 3.0 T MR scanner (ACHIeva 3.0 T Quasar Dual; Philips Medical Systems) using an 8ch SENSE head coil. For anatomical MRI, a 3D fastfield echo T1-weighted sequence was used (TR/TE = 7.0/3.3 ms, FOV = 256 mm, 200 slices, acquisition voxel size = 1.00 × 1.00 × 1.00 mm, and a slice thickness = 1.0 mm). Cortical thickness was measured using FreeSurfer software (version 6.0 (https://surfer.nmr.mgh). harvard.edu, accessed April 16, 2023). This software was used to evaluate the cortical thickness using MRI data, allowing for cortical reconstruction of the whole brain. This allowed the individual T1-weighted images to be registered in the Talairach standard space with the skulls stripped, in addition to correcting for small motions. The cortical surface of each hemisphere was inflated to an average spherical surface to locate both the pial surface and white matter/gray matter boundary. Cortical thickness was measured based on the shortest distance between the pial surface and white matter/gray matter boundary at each point across the cortical mantle. Both the cerebellar surface and white matter/gray matter boundary were positioned. For quality control of the preprocessed images, white matte/gray matter boundaries and segmentation of the subcortical structures were visually checked by S.T. and K.Y. The cerebral cortex was divided into 34 regions in each hemisphere according to the Desikan-Killiany cortical labeling protocol, and the mean cortical thickness of each region was calculated^52^.

### Assessment of pulmonary function

Pulmonary function was assessed using the CHESTAC-8800 DN system (Chest Ltd., Tokyo, Japan). The measurement method was based on the recommendations of the American Thoracic Society/European Respiratory Society^53^. The severity of COPD was assessed using the GOLD classification^54^.

### Assessment of physical activity

A physical activity meter with triaxial acceleration (Active style ProHJA-750C; Omron Ltd., Tokyo, Japan) was fitted for 2 weeks. The amount of physical activity was evaluated by selecting the last 3 days from the measurement days, excluding holidays and rainy days, and using the average value of the activity time. We measured metabolic equivalents (METs) and calculated exercise (EX), which is the value of metabolic equivalents multiplied by their duration (METs × hours per day), as previously reported^9,55,56^.

### Examination of cortical thickness and physical activity

Cortical regions that were thinner than those of healthy subjects and significantly correlated with KCL were correlated with EX.

### Statistical analysis

All statistical analyses were performed using the JMP Pro version 16.00 (SAS Institute Inc., Cary, NC, USA). Comparisons of the characteristics and cortical thickness between the COPD and healthy groups were performed using the Mann–Whitney U test. Data are expressed as mean ± standard deviation unless otherwise indicated. Correlations between the frailty score and cortical thickness were examined using the Spearman's rank correlation coefficient. Correlations between the EX and cortical thickness were examined in a similar manner. Spearman's partial correlation analysis with age, MoCA-J score, BMI, EX, % FEV1, and sex as covariates was performed to identify significant correlations. We used a dummy coding scheme for the binary variable when conducting partial correlations, with sex as a covariate. Spearman's rank correlation was used to examine which KCL questions correlated significantly with cortical thickness reduction. Statistical significance was set at *p* < 0.05.

### Supplementary Information

Supplementary Tables.

Supplementary Figure S1.

Supplementary Figure S2.

Supplementary Figure S3.

## Data Availability

The datasets used and/or analyzed in the current study are available from the corresponding author upon reasonable request.
