# Supplementary material for: Correlation between frailty and reduction in cortical thickness in patients with chronic obstructive pulmonary disease
Source: Sci Rep. 2024 Mar 13;14:6106. doi: 10.1038/s41598-024-53933-0 (PMC10937661; doi:10.1038/s41598-024-53933-0)
Supplement: Supplementary file 1 — Supplementary Tables. [file 41598_2024_53933_MOESM1_ESM.docx]

Supplementary Table S 1.

1. Partial correlation between frailty and cortical thickness adjusting for age as a covariate.

|  | **Healthy**  **(n=20)** | | | **COPD**  **(n=40)** | |
| --- | --- | --- | --- | --- | --- |
|  | **KCL** | | | | |
|  | ***ρ*** | ***p*** | ***ρ*** | | ***p*** |
| **Left** |  |  |  | |  |
| Superior　frontal | -0.0532 | 0.8289 | -0.5308 | | 0.0005 |
| **Right** |  |  |  | |  |
| Superior　frontal | -0.0151 | 0.9511 | -0.5361 | | 0.0004 |
| Insula | 0.3326 | 0.1642 | -0.4916 | | 0.0015 |
| Para hippocampal | 0.1388 | 0.5708 | -0.4470 | | 0.0043 |
| Post　central | -0.2730 | 0.2581 | -0.3357 | | 0.0367 |
| Rostral middle frontal | 0.0790 | 0.7478 | -0.3472 | | 0.0303 |
| Supra　marginal | 0.4483 | 0.0542 | -0.3434 | | 0.0333 |

*COPD*: chronic obstructive pulmonary disease, *KCL*: Kihon Checklist.

1. **Partial correlation between frailty and cortical thickness adjusting for age, the MoCA-J score and BMI as covariates.**

|  | **Healthy**  **(n = 20)** | | | **COPD**  **(n = 40)** | |
| --- | --- | --- | --- | --- | --- |
|  | **KCL** | | | | |
|  | ***ρ*** | ***p*** | ***ρ*** | | ***p*** |
| **Left** |  |  |  | |  |
| Superior　frontal | -0.2568 | 0.3197 | -0.5377 | | 0.0006 |
| **Right** |  |  |  | |  |
| Superior　frontal | -0.1471 | 0.5731 | -0.5320 | | 0.0007 |
| Insula | 0.2743 | 0.2867 | -0.4892 | | 0.0021 |
| Para hippocampal | 0.2204 | 0.3953 | -0.4499 | | 0.0052 |
| Post　central | -0.1839 | 0.4798 | -0.3547 | | 0.0312 |
| Rostral middle frontal | 0.0965 | 0.7125 | -0.3417 | | 0.0385 |
| Supra　marginal | 0.5424 | 0.0245 | -0.3556 | | 0.0308 |

*MoCA-J*: Japanese version of the Montreal Cognitive Assessment, *BMI*: body mass index.

**Supplementary Table S2.**

1. **Correlation between cortical thickness and the EX in patients with COPD and healthy participants.**

|  | **Healthy**  **(n = 20)** | | | **COPD**  **(n = 40)** | |
| --- | --- | --- | --- | --- | --- |
|  | **EX** | | | | |
|  | ***ρ*** | ***p*** | ***ρ*** | | ***p*** |
| **Left** |  |  |  | |  |
| Superior　frontal | 0.3402 | 0.1671 | 0.3023 | | 0.0614 |
| **Right** |  |  |  | |  |
| Superior　frontal | -0.0114 | 0.9643 | 0.3569 | | 0.0257 |
| Insula | 0.5315 | 0.0232 | 0.3690 | | 0.0208 |
| Para hippocampal | 0.2570 | 0.3033 | 0.6071 | | ＜.0001 |
| Post　central | 0.0857 | 0.7353 | 0.0933 | | 0.5720 |
| Rostral middle frontal | 0.0506 | 0.8420 | 0.3897 | | 0.0142 |
| Supra　marginal | 0.4303 | 0.0746 | 0.2973 | | 0.0660 |

*EX*: exercise.

1. **Partial correlation between frailty and cortical thickness with age, the MoCA-J score, BMI and the EX as covariates.**

|  | **Healthy**  **(n = 20)** | | | **COPD**  **(n = 40)** | |
| --- | --- | --- | --- | --- | --- |
|  | **KCL** | | | | |
|  | ***ρ*** | ***p*** | ***ρ*** | | ***p*** |
| **Left** |  |  |  | |  |
| Superior　frontal | -0.3555 | 0.1766 | -0.4920 | | 0.0023 |
| **Right** |  |  |  | |  |
| Superior　frontal | -0.1402 | 0.6045 | -0.4672 | | 0.0041 |
| Insula | 0.2908 | 0.2745 | -0.4017 | | 0.0152 |
| Para hippocampal | 0.1263 | 0.6411 | -0.2905 | | 0.0856 |
| Post　central | -0.2572 | 0.3362 | -0.4233 | | 0.0103 |
| Rostral middle frontal | 0.0742 | 0.7847 | -0.2277 | | 0.1817 |
| Supra　marginal | 0.5267 | 0.0361 | -0.2926 | | 0.0833 |

*KCL*: Kihon Checklist, *MoCA-J*: Japanese version of the Montreal Cognitive Assessment, *BMI*: body mass index.

**Supplementary Table S3**. **Partial correlation between frailty and cortical thickness with age, the MoCA-J score, BMI and sex as covariates.**

|  | **Healthy**  **(n = 20)** | | | **COPD**  **(n = 40)** | |
| --- | --- | --- | --- | --- | --- |
|  | **KCL** | | | | |
|  | ***ρ*** | ***p*** | ***ρ*** | | ***p*** |
| **Left** |  |  |  | |  |
| Superior　frontal | -0.2661 | 0.3191 | -0.5179 | | 0.0012 |
| **Right** |  |  |  | |  |
| Superior　frontal | -0.1476 | 0.5853 | -0.5050 | | 0.0017 |
| Insula | 0.2748 | 0.3030 | -0.4776 | | 0.0032 |
| Para hippocampal | 0.2576 | 0.3354 | -0.4264 | | 0.0095 |
| Post　central | -0.1840 | 0.4951 | -0.3270 | | 0.0516 |
| Rostral middle frontal | 0.0975 | 0.7195 | -0.3112 | | 0.0647 |
| Supra　marginal | 0.5482 | 0.0279 | -0.3277 | | 0.0511 |

*KCL*: Kihon Checklist, *MoCA-J*: Japanese version of the Montreal Cognitive Assessment, *BMI*: body mass index.
