# Supplementary figures and images for: Correlation between frailty and reduction in cortical thickness in patients with chronic obstructive pulmonary disease
Source: Sci Rep. 2024 Mar 13;14:6106. doi: 10.1038/s41598-024-53933-0 (PMC10937661; doi:10.1038/s41598-024-53933-0)

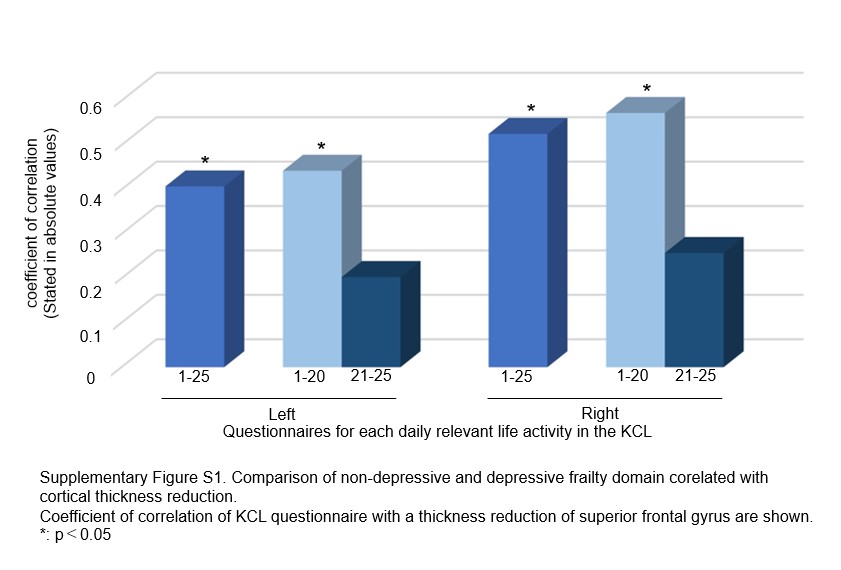

Supplement: Supplementary file 2 — Supplementary Figure S1. [file 41598_2024_53933_MOESM2_ESM.jpg]

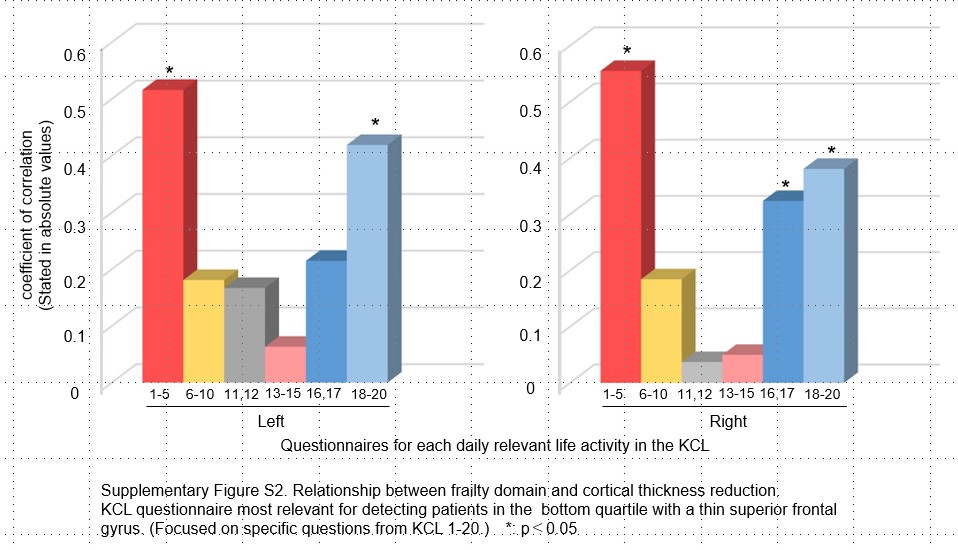

Supplement: Supplementary file 3 — Supplementary Figure S2. [file 41598_2024_53933_MOESM3_ESM.jpg]

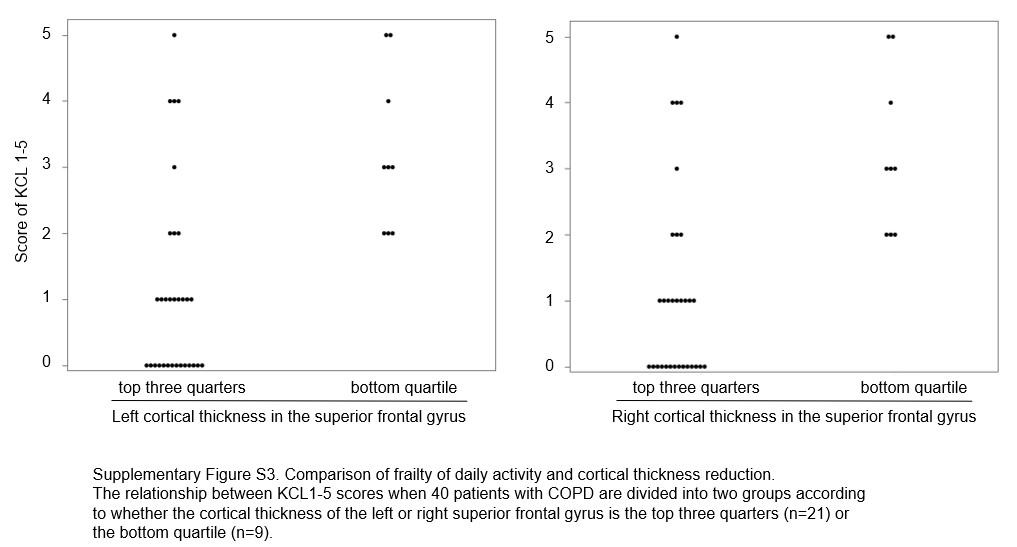

Supplement: Supplementary file 4 — Supplementary Figure S3. [file 41598_2024_53933_MOESM4_ESM.jpg]
